# Institutional Logics in the UK Construction Industry’s Response to Modern Slavery Risk: Complementarity and Conflict

**DOI:** 10.1007/s10551-023-05455-4

**Published:** 2023-06-07

**Authors:** Christopher Pesterfield, Michael Rogerson

**Affiliations:** 1 University of Bristol, Howard House, Queens Avenue, BS8 1SD Bristol, UK; 2 Surrey Business School, University Of Surrey, Alexander Fleming Rd, Guildford, GU2 7XH UK

**Keywords:** Modern slavery, Construction industry, Institutional logics

## Abstract

There is a growing understanding that modern slavery is a phenomenon ‘hidden in plain sight’ in the home countries of multinational firms. Yet, business scholarship on modern slavery has so far focussed on product supply chains. To address this, we direct attention to the various institutional pressures on the UK construction industry, and managers of firms within it, around modern slavery risk for on-site labour. Based on a unique data set of 30 in-depth interviews with construction firm managers and directors, we identify two institutional logics as being integral to explaining how these companies have responded to the Modern Slavery Act: a market logic and a state logic. While the institutional logics literature largely assumes that institutional complexity will lead to a conciliation of multiple logics, we find both complementarity and continued conflict in the logics in our study. Though we identify conciliation between aspects of the market logic and the state logic, conflict remains as engagement with actions which could potentially address modern slavery is limited by the trade-offs between the two logics.

## Introduction

Modern slavery continues to be an important ethical issue for managers and risk for organizations (Cousins et al., 2020). These risks come in the form of labour abuses which are found in businesses, either directly or in their supply chains. Modern slavery is increasingly understood as a continuum of exploitation, reflecting the complex nature of the practices that are found in different contexts (Boersma & Nolan, 2022; Rioux et al., 2020). For example, at the more severe end there is “the exploitation of a person who is deprived of individual liberty anywhere along the supply chain” (Gold et al., 2015, p.487). The less severe practices in the continuum might not involve the absence of liberty, in the sense of direct coercion, but can include issues such as non-payment or under-payment of workers. This range is represented, respectively, by recent cases of widespread abuses of Uyghurs in Xinjiang, China (Kriebitz & Max, 2020) and at Boohoo’s supplier factories in the UK (Stevenson, 2022). These have highlighted risks at home and abroad and brought increased scrutiny on firms.

In the UK, legislation on the issue has taken the form of the Modern Slavery Act 2015 (MSA), which intends to drive responsible labour practices down supply chains through the transparency in supply chains clause found in Sect. 54. This obliges annual publication of a modern slavery statement by firms with a turnover above £36m and encourages those firms to disclose the actions they have taken to address modern slavery risks in their supply chains. In doing so, firms are expected to use their influence as buyers to compel their suppliers to comply with MSA and address risks themselves. MSA is therefore designed to cascade improvements in transparency down supply chains to the lower tiers, where focal firms have limited visibility. The aim of this mechanism is for best practice to advance firm reporting year on year, improving both industry and field performance (Rogerson et al., 2020). However, since this process was not codified in law, reporting under the Act has remained consistently poor (Pinnington et al., 2022).

The enactment of MSA was followed by the development of both academic (e.g. Flynn, 2020; Flynn & Walker, 2021; Meehan & Pinnington, 2021; Schaper & Pollach, 2021; Stevenson & Cole, 2018) and industry (e.g. BHRRC, 2017, 2021; Ergon, 2018) research into disclosure under the Act. The literature has thus far largely focussed on measuring compliance and a body of scholarly research has yet to emerge which explains organizational behaviour either in response to the legislation or to the wider risks of modern slavery in supply chains.

We address this gap in the literature by investigating how the UK construction industry understands and prioritizes modern slavery risks. The sector was chosen because it is high risk for modern slavery (Crates, 2018; GLAA, 2020) and has received only limited scholarly attention. This paper focusses on how industry actors have responded to the presence of these exploitative practices considering both the coercive pressure of the legislation and the normative pressure associated with the ethical issue of modern slavery (Wray-Bliss & Michelson, 2021). This occurs within the context of an industry where work is awarded principally on price (Hartmann & Caerteling, 2010), in a highly competitive sector with low enough profit margins that the Confederation of British Industry, a trade body representing around 200,000 British businesses, suggests that the average margin at the industry’s largest firms is actually negative (CBI, 2020).

To better comprehend firm behaviour around modern slavery, we draw on institutional theory, which helps to explain how pressures on firms are understood and translated into action. Institutions are societies’ formal rules and taken-for-granted practices underpinning how organizations are required to act (Campbell, 2006). Those organizations failing to conduct themselves within the bounds of these accepted behaviours risk their legitimacy (Palazzo & Scherer, 2006). Organizations are therefore under constant pressure to adapt to the demands in their institutional environments.

In order to understand responses to societal institutions, scholars have increasingly turned to institutional logics. Institutional logics are “material practices and symbolic constructions” that represent the organizing principles of society and that are “available to organizations and individuals to elaborate” (Friedland & Alford, 1991, p.248). Shared institutional logics, stemming from how managers recognize and process the institutional pressures on them and their organizations, enable organizations to obtain and maintain legitimacy through responding to institutional pressures with actions acceptable to stakeholders (Palazzo & Scherer, 2006). Institutional logics therefore offer researchers a framework for investigating how actors understand and react to the institutional pressures on them. Using institutional logics as a tool for such understanding, we ask the research questions: *What are the institutional logics affecting UK construction industry responses to modern slavery risk, and how are they reconciled to produce mitigation strategies?”* Within this question, mitigation strategies are understood as the measures implemented in response to modern slavery risks.

We respond by drawing on a series of 30 in-depth semi-structured interviews, an established method for identifying and analysing institutional logics and organizational behaviour (Corbett et al., 2018; Nicholls & Huybrechts, 2016), with managers and directors of principal construction firms (i.e. the main contractors responsible for construction project delivery). We use pattern matching to match our data against two institutional logics that are identified in the literature: a market logic and a state logic. We find that these two logics both complement one another and compete for primacy. Competing logics create contradictory pressures on firms, creating potential points of conflict between organizations and their stakeholders (Reay & Hinings, 2009). We find, contrary to the uneasy conciliation between logics advanced in the literature (e.g. Savarese et al., 2020; Lounsbury et al., 2021), an irreconcilable contest between aspects of the logics in the industry. We therefore present evidence of both complementarity and conflict between the two logics, with the resulting relations between the two logics limiting firm actions in response to modern slavery risks.

Our study makes three contributions. First, we contribute to the nascent literature on how firms are addressing modern slavery risks in their operations. By focussing on the institutional logics around modern slavery in the construction industry, we highlight how logics both compel and undermine action to address modern slavery in the local labour supply chains of principal contractors.

Second, we contribute to the literature on institutional logics by suggesting boundary conditions which question the existing assumption that tensions between competing logics, particularly on issues of sustainability, can be settled in a way that emphasizes facets of multiple logics. While aspects of the state logic relating to legal compliance complement the market logic, the latter also prevents firms from adequately addressing modern slavery risks in their labour supply, creating an irreconcilable conflict between the logics we identify in the industry.

Third, we contribute to the literature on the construction industry. We highlight the importance of the dual role of government as construction client and legislator, creating regulation that is difficult to meaningfully comply with because of the low-cost approach to tendering. We suggest that a sector known to be a high risk for modern slavery is unable to take meaningful action to mitigate risks because clients—including government—are unwilling to pay for the work that is required to adequately manage modern slavery risks on site.

The remainder of our paper is structured thus: in the following section we review the literature on modern slavery and MSA, the presence of modern slavery practices in the UK construction industry, and institutional logics. In the third section, we describe our “Methodology”. Section 4 lays out our analysis and “Findings”. Section 5 is a “Discussion” of our findings in reference to the relevant literatures. Finally,  in section 6 we explain the “Implications and conclusions” of our study for researchers, managers, and policymakers.

## Literature Review

### Modern Slavery

Despite increasing attention from policymakers around the world on the subject, a cohesive body of scholarly research has yet to emerge on modern slavery in the management literatures, a broad area recently—and aptly—described as a ‘sorry non-field’ (Caruana et al., 2021). Beyond that critique of extant research, however, there is a discernible thematic development in the literature as the thinking of those scholars engaged with the subject matures.

The literature to date can be broadly classified into three themes. The first publications on the subject were conceptual, describing the conditions in which modern slavery can thrive (e.g. Crane, 2013); the challenges presented to supply chain management by recognition—legally and phenomenologically—of the issue (Gold et al., 2015; New, 2015); or the many and various failings inherent in the legislation (Islam & van Staden, 2021; Pesterfield, 2021), particularly resulting from government compromising the legislation before it was enacted (LeBaron & Rühmkorf, 2017; 2019).

The failings of MSA lead to a second theme: that of the (poor) level and (limited) nature of compliance with MSA. Even organizations operating in sectors known to have a high risk of modern slavery in their supply chains, such as agriculture (Phillips & Trautrims, 2018) and apparel (Stevenson & Cole, 2018) have failed to even comply with the legislation, let alone publish evidence of meaningful action to address threats to vulnerable people in their supply chains. This can be attributed largely to a focus on developing new policies and strengthening contract terms rather than focussing on the root of the problem in supply chains (Flynn & Walker, 2021). This superficial approach is mirrored in the literature, where several studies have focussed on the underlying factors predicting higher levels of compliance, without regard for organizational action taken to tackle risks (e.g. Flynn, 2020).

As scholars have sought to understand the underlying processes behind efforts to address modern slavery risks in response to MSA, a third theme has emerged. Only in this last theme do we begin to see the ethical choices made by and around managers regarding modern slavery. Both the food and tobacco industries are highlighted in the literature as featuring deliberate efforts to circumvent the spirit of the law (Monciardini et al., 2021). Pinheiro et al. (2019) highlight the difficulties for focal firms in cascading the law down their supply chains to locations that are not themselves within the scope of MSA, finding performative compliance with customer demands on supply chain ethics. Evidence is also emerging, in the UK construction industry specifically, of how different framings of modern slavery have prevented substantial action being taken on the issue (Gutierrez-Huerter O et al., 2021).

Despite this theme having behavioural aspects at its core, there remains a focus on legal compliance and the issues of managing—or choosing not to manage—the opacity in international supply chains. The role of the complex nature of globalized supply chains is now taken for granted in discussions of modern slavery (e.g. Meehan & Pinnington, 2021; Stevenson, 2022; Voss et al., 2019). Even the architect of MSA, Theresa May, stated early in her tenure as UK Prime Minster that modern slavery was a “vile and systematic *international* business model” (May, 2016, *emphasis added*). Risks in firms’ immediate operations within the UK remain largely ignored (Garbers, 2022), and we therefore situate our study within the third theme we identify—actions and choices taken by managers—with a specific emphasis on UK operations as a context.

### Modern Slavery in the Construction Industry

Where conversations around modern slavery risks within the UK occur, attention is drawn to the apparel and textiles industry (e.g. Benstead, 2018). While apparel is a known high-risk sector (Stevenson, 2022), the Gangmasters and Labour Abuse Authority, an organization partnered with UK police forces to investigate labour exploitation, focusses on the construction industry in its most recent report (GLAA, 2020). Modern slavery charity Unseen (2019) lists construction as one of the top three sectors for modern slavery risk in the UK. However, there is limited literature either on the construction industry’s response to MSA or responses that look specifically at responding to modern slavery risks within the UK. The little literature that does elucidate the industry’s approach to dealing with modern slavery risk (e.g. Trautrims et al., 2021; Gutierrez-Huerter O et al., 2021) also includes the complexity of supply chains among the major risk factors. An important gap therefore remains in investigating more localized risks.

The UK construction industry, and specifically on-site labour in the UK, is an interesting context for our study because it is a high-risk sector for modern slavery (GLAA, 2020). One reason for this is the extensive use of subcontracting, particularly for blue-collar work, which has led to the predominance of ‘hollowed out’ firms at the top of supply networks which focus primarily or solely on white-collar management work (Alberti & Danaj, 2017). It is common practice for subcontractors who are awarded work by a principal contractor to then further subcontract work to other subcontractors or labour agencies, which may then further subcontract out elements of this work. As a result, there can be thousands of firms on a single project, and a complex, opaque supply chain of contractors (Manu & Knight, 2020). One consequence of this is a revolving door of workers, with turnover on some sites being up to 200% (ILO, 2001).

Additionally, there is a widespread use of non-standard employment (NSE) practices, which results in a limited number of directly employed workers. NSE includes bogus self-employment, where conditions do not meet normal criteria for self-employment such as choosing working hours and pay (Behling & Harvey, 2015). As Clarke (2006, p.249) puts it, the combination of self-employment and subcontracting has resulted in “extreme fragmentation” within the sector, with about “170,000 private contracting companies of which about 40% are private one-person firms and over 93% have fewer than 13 employees”. This fragmentation means principal contractors often lack knowledge of what is happening even in the supply chain tiers immediately around them (Pala et al., 2014). All of this occurs in an unregulated industry where the GLAA has no jurisdiction.

Migrant workers—in some cases undocumented—are also prevalent in the sector, and background checks are not always in place (Crates, 2018). As such, construction presents an opportunity for organized crime (Zielinski, 2019), counterfeit Construction Skill Certification Scheme cards which are often used to verify workers’ identity (CITB, n.d.), and links between fraud in construction certification to people trafficking and modern slavery (Ali, 2018). Despite the ‘hidden’ nature of modern slavery, there have been several documented cases in the sector, with individuals found working on a site run by one of the UK’s largest contractors under coercion for only £2 per day and living with their exploiters in squalid conditions (Garner-Purkis, 2019). A 2021 investigation culminated in the arrest of 13 men for trafficking over 50 individuals and placing them onto construction sites under exploitative conditions and without proper payment (Weinfass, 2021). Finally, physical and verbal abuse of workers and non-payment issues has been found for migrant workers in London (FLEX, 2018), as well as deductions for equipment or housing leading to wages well below the legal minimum (Taylor & Addley, 2008).

To date, there are only three studies which have attempted to explain the construction industry’s response to these risks. Jones and Comfort (2022) focus on the content of modern slavery statements of UK housebuilders, and therefore focusses only on what companies say they have done in public disclosures. Trautrims et al., (2021, p.290) produce insight into the actions taken, finding that companies have “focussed on the integration of anti-slavery measures in existing processes”, and that they have “avoided substantial investments in new infrastructure”. In other words, any changes that have been made thus far to how construction companies mitigate against modern slavery risks have been superficial. Similarly, Gutierrez-Huerter O et al. (2021) argue that the only real change has been to rhetoric rather than action within the sector.

### Institutional Logics

As a result of the limited insights produced, we know little of the competing pressures on even those managers wishing to make ethical decisions and develop a robust response to modern slavery risk. It is for this reason that we use an institutional logics approach which can help to make sense of how managers understand these competing pressures (Thornton & Ocasio, 1999) and what this means for how companies’ risk mitigation strategies are shaped, i.e. firm understanding of, and responses to, the pressures under which they are operating (Nicholl & Huybrechts, 2016). Institutional logics are distinguishable from particular behaviours by nature of their being the broader underlying “supraorganizational patterns of activity” (Friedland & Alford, 1991, p. 232). That is, logics inform behaviour. In relation to how managers respond to MSA, there are two key logics: a market logic and a state logic.

#### Market Logic

In much of the business literature on institutional logics, market logic speaks to decision making aimed at maximizing economic returns (Thornton et al., 2012). Driven by a profit imperative, the market logic is evident in organizational efforts to enhance efficiency and save costs (Dahlmann & Grosvold, 2017). The focus of the market logic on profit maximization brings it into competition with other logics with which it is seen as incompatible. Such discord is particularly the case when the market logic is forced to compete with logics derived from pressures to make firms more sustainable, since actions aimed at sustainability incur costs which have traditionally been viewed as not contributing to profitability (Glavas & Mish, 2015). The market logic is therefore key to understanding company behaviour for the present study because MSA is intended to harness competitive market forces to increase transparency, and thus reduce instances of modern slavery (HM Gov, 2015). This process is intended to function in the highly competitive low-margin market within which construction companies operate.

#### State Logic

The state logic pertains firstly to individuals’ understanding of societal pressures to conform with legislation and regulations, i.e. with the coercive interventions of government (Yin & Jamali, 2021). Such regulations usually set minimum standards of behaviour to which organizations are expected to conform. However, the state logic also produces a normative pressure—which is implied in MSA (Rogerson et al., 2020)—for firms to contribute to societal goals beyond the narrow remit of legislation. As such, the state logic refers to the maintenance of order and general acquiescence to accepted principles (Arena et al., 2018). The orientation of the state, therefore—represented by the state’s aims and goals set out in law and also, increasingly, in the form of general guidance and counsel relating to formal regulation—is an important driver of organizational behaviour (Greenwood et al., 2010). While regulation exists to set concrete standards with which organizations must comply, however, an openness to plurality remains outside such rules for organizations to interpret their roles and responsibilities beyond minimum standards. Such flexibility is key in, for example, considerations of the role of the corporation in society and the ethical questions around the broad topic of corporate responsibility (Westermann-Behaylo et al., 2014). Consequently, the state, as the producer of MSA, is a key institution for this study.

#### Competing Logics

New logics arise through institutional shocks or the emergence of new ‘social facts’, either suddenly—through the enactment of new legislation—or over time, through the purposeful effort of some actors. New logics create tension with existing logics (Ocasio et al., 2015). Early scholarship on institutional logics pointed to the existence of a dominant logic, i.e. that fields were organized according to the demands of the most powerful actors (Reay & Hinings, 2009). However, institutional logics scholars now believe that this has been superseded by the acceptance of institutional and organizational dynamism (Besharov & Smith, 2014; Lounsbury et al., 2021). The result is a body of work in which the competition between logics results in an uneasy conciliation of influences which continue to vie for primacy (Waldorff et al., 2013). In some contexts, a natural conciliation of logics occurs because context requires that balance, for instance between social good and market logic in social enterprises (Savarese et al., 2020). However, in other settings, such as healthcare, a medical professionalism logic can co-exist with a market logic not though conciliation but through the management of the conflict between the logics (Reay & Hinings, 2009). Thus, despite the dominance of the market logic, multiple logics continue to co-exist as individuals within organizations manage the tensions between logics (Dahlmann & Grosvold, 2017; McLoughlin & Meehan, 2021).

The institutional logics perspective therefore has clear implications for understanding how organizations deal with competing institutional pressures. Consequently, the theory has been employed to explain and understand a diverse range of (un)ethical behaviours in various areas of corporate responsibility which we might consider analogous to our study. The literature on institutional logics has found conciliation of logics but has yet to gain an appreciation for those cases where logics cannot be reconciled. The question of how the competing demands of rival institutional logics are interpreted by organizations—and how those organizations navigate those competing demands—has begun to be investigated, but the conflicting institutional pressures on firms have received insufficient attention (Lee & Lounsbury, 2015). Furthermore, despite the explanatory power of institutional logics, specific calls to employ logics to better understand the decision making and processes underlying organizational responses to modern slavery risks (e.g. Gümüsay et al., 2020; Phung & Crane, 2018) remain unanswered. Similarly, scant research has been published on the construction industry and modern slavery using a theoretical lens.

## Methodology

### Methods

We investigated the research questions—*what are the institutional logics affecting UK construction industry responses to modern slavery risk, and how are they reconciled to produce mitigation strategies?—*through semi-structured interviews with practitioners working for principal contractors in the construction industry. Doing so allowed us to generate data on how practitioners make sense of, and attempt to reconcile, the expectations and pressures they face in developing responses to the presence of modern slavery. Concentrating on a single industry, and specifically on the principal contractors that sit at the top of labour supply chains, allows us to contribute to a research gap highlighted by Flynn and Walker (2021, p.307), who point to the need for “in-depth case analyses into how focal firms manage the organisational challenges of de-risking their supply chains from modern slavery”.

Semi-structured interviews were used because they are able to facilitate “a strong element of discovery” (Gillham, 2007, p.72), which is necessary for an industry which has received little attention from modern slavery scholars. The senior positions of the participants—detailed below—made it necessary to use a method that is most effective for elites who hold a high level of knowledge about their sector and their own company’s approach and practices within this (Lancaster, 2017). As such, interviews allowed us to “produce situated knowledge” (Mason, 2018, p.113), and to enhance participants’ ability to express themselves as fully as possible within the structured element (Aberbach & Rockman, 2002).

### Sample

We conducted 30 semi-structured interviews with managers and directors working in procurement, supply chain, and sustainability roles within principal contractors (see Appendix 1). We selected professionals in these roles because their departments are responsible for managing modern slavery risks. Principal contractors were chosen due to the position they occupy at the top of labour supply chains, bidding for work from clients and holding responsibility for the delivery of the project. Through competitive tender, these contractors choose the companies they will outsource work to. Such arrangements give them significant influence and leverage over the ways in which a project is managed, which parts are outsourced, and in setting standards for how work is carried out (Van Buren et al., 2021). They are also invariably the company in the supply chain with the largest turnover, and consequently have considerable resource. The position this primacy gives them in their supply chains also makes them, per the spirit of MSA, responsible for driving anti-modern slavery actions down into their supply chains (Rogerson et al., 2020).

Our sample was produced using a purposive sampling method. This allowed us to select participants in specific roles in specific companies, and to generate suitable data to “information-rich cases” (Palinkas et al., 2015, p.534). In the first instance, participants were identified through The Construction Index’s list of the top 100 companies by turnover, and then contacted through LinkedIn or email. In response to concerns over recruiting through social media sites such as LinkedIn (Bhatia-Lin et al., 2019; Gelinas et al., 2017; Spino, 2019) we treated this form of recruitment as commensurate with more traditional forms by sharing no personal or sensitive information, and ensuring participants were aware they were being contacted for the purposes of research. Sampling continued beyond the point of saturation, often referred to as the “gold standard” for assuring the quality, or validity, of qualitative data (Saunders et al., 2018, p.1894). Saturation was judged in accordance with a point made by Braun and Clarke (2021, p.205), that meaning does not “reside in data”, but rather that the researchers made an informed “interpretative, situated, and pragmatic judgement” (ibid, p.211) about when sufficient data had been collected.

### Data Collection

We focussed our questions on modern slavery risks relating to labour supply chains for on-site labour due to the leverage principal contractors have over the firms they subcontract work to. Additionally, all the firms in a labour supply chain operate within the UK and are required to adhere to all relevant domestic labour laws including MSA. Within this, our questions explored the key drivers or inhibitors for practitioners’ addressing modern slavery risks. Interviews were conducted and recorded using videoconferencing apps, partly due to the convenience they offer (Janghorban et al., 2014), but also in large part because the interviews took place shortly after the outbreak of the coronavirus (COVID-19) pandemic. Due to the necessity of protecting confidentiality, company turnover is not reported, which would make certain companies, and therefore participants, potentially identifiable. Instead, an approximation of the firm size is presented within the context of the top 100 companies in the UK by turnover.

### Data Analysis

The data were analysed using pattern matching, which is a method for comparing existing concepts, in this case logics, with empirical data (Bouncken et al., 2021; Sinkovics, 2018). As Bouncken et al., (2021) highlight, pattern matching is not a linear process of simply identifying data that have been collected; it is instead an iterative process of moving between theory and empirical data. As such, the below details the steps we followed to produce our findings and analysis.

First, it was necessary to identify relevant “ideal types” from existing literature, in order to then explore to what extent empirical data can be matched to each ideal type (Reay & Jones, 2016, p.446). This provided a systematic way of both grounding our analysis in existing logics, in addition to aiding judgements about how our data conformed to or contradicted those logics identified in the literature. The immediate result of this literature review was the identification of several logics that had potential relevance for our analysis, including state, market, compliance, and responsibility logics.

Once we had identified these logics, the next step involved coding interview transcripts using Nvivo 12 software, and through this process the data were thematically categorized to “reveal the existing underlying meanings and thus identify patterns of behaviours and beliefs associated with particular logics” (Reay & Jones, 2016, p.449). Once this was complete, we conducted the process of matching data to the logics we had identified in the literature. In other words, we answered the question of whether in our data we found evidence of, say, the market logic in the form of practitioners suggesting profit was a consideration for how they have acted in addressing modern slavery. We carried out this process for each logic.

Matching between logics and against data, it became evident that the institutions of the market and the state were the key sources of the pressures the firms in our sample are under. These sources, and their associated logics, act as normative drivers of behaviour (Nicholls & Huybrechts, 2016) and relate, respectively, to the competitive conditions within which construction contractors operate, and how MSA is intended to function through harnessing market forces to bring about a reduction in modern slavery. The other logics which were initially identified from the literature were ultimately discarded due to their overlap with state and market logics. For example, compliance logic was subsumed into both state and market logics because both the latter logics contain drivers for compliance with MSA, as identified in the findings below. This negated the need to include compliance as a separate logic. The same applied to other logics identified earlier in the process, resulting in the final two of market and state.

Further immersion in the data was undertaken to develop an understanding of how the two logics create pressures on managers; how these pressures were experienced; and finally, how the two logics are perceived to interact by participants and what this means for how they respond to MSA. In this stage of data analysis, we found two key dynamics through which the logics interact: complementarity and conflict. As detailed below in our findings after each logic has been introduced and explained, the specific ways in which the logics are found by managers to either complement each other or come into conflict directly impacts the decisions managers made.

## Findings

This section reveals the extent to which our data match the two logics identified in the pattern matching process—the market logic and the state logic—as potentially impacting responses to the presence of modern slavery in the construction industry. First, we explain how our data match each of these logics, and how the pressures which characterize the logics are understood by respondents. We then analyse how these logics interact with each other.

### Market Logic

In our data, the market logic is, unsurprisingly, present as one of the key factors driving decisions made within businesses in response to MSA. In addition to the pressure to operate with low costs in a low-margin sector, the market logic comes in three forms: reputational risks; competitor behaviour; and client expectations.

First, reputational considerations constitute an important element of what motivates firms to respond to MSA. We find in our data the suggestion that sufficient awareness has been raised of modern slavery that media reporting of any on-site incidents has become a concern for large contractors, whether this is national or construction-specific media.

*I won’t lie, half of it is we just don’t want the bad press from it… I know that sounds wrong because it should be about doing the right thing and that is there, but I’m not gonna fluff it up and pretend that there isn’t a commercial reason for doing these things.* (Participant 12)

Second, competitor behaviour also impacts decision around which mitigation measures to adopt or use. Contractors are aware of what their competitors are doing, and assume their competitors are paying attention to their own actions. As such, there is a motivation to implement similar responses to competitors in order not to fall behind, but there is also little or no market-based motivation to implement more substantial measures than competitors.

*It’s difficult to get a competitive advantage probably over the tier one main contractors because we’re all doing very similar stuff around [modern slavery] and we’re all using a very similar supply chain.* (Participant 22)

Third, clients are integral to the market conditions through which contractors operate because they establish the conditions the latter have to meet in order to bid for work. This applies to numerous areas, including modern slavery, where certain minimum standards must be met.

*You wouldn’t get to play in that big space if you didn’t have [modern slavery policies] in place because the clients would say “you don’t look as if you’ve got enough in place here”.* (Participant 19)

These three elements of the market logic demonstrate how addressing modern slavery is necessary to avoid reputational damage and secure work, but they also reveal that market logic places a limit on actions taken because there is little or no advantage to be gained over competitors.

### State Logic

We also matched our data against the state logic, which for the present context refers not only to the obligations placed on firms stemming from MSA, but also normative expectations to act responsibly, which MSA is also designed to engender.

Our data reveal that the state logic is present through practitioners identifying MSA as a key catalyst for actions that have been taken, both in the sense that there was a new legal obligation, but also through the awareness the Act raised of the issue.

*It’s certainly something we would be more wary of, more aware of, than we were prior to that Act and probably shows where the government gives it a little bit of a push and support then it’s suddenly something that’s new, that’s perhaps got a little bit more traction.* (Participant 5)

*Our maturity has grown as part of that regulatory or mandatory reporting. The first year of getting to grips with reporting was the kind of actually, is this really an issue? Modern slavery really exists? And having to go through that education phase to waking up to, wow, crikey, okay, yeah this could be really quite a big issue.* (Participant 10)

These comments reveal that, beyond the state’s ability to bring about change through legislation, this same mechanism was the starting point for practitioners learning about modern slavery. Our data also evidence the normative aspect of MSA, with some suggesting their intention is to not only comply with the Act by producing a modern slavery statement, but also to make attempts to reduce modern slavery risks. As one Ethical Procurement Manager put it:

*I'll be honest, we didn't look at this before the Modern Slavery Act, it is the Modern Slavery Act that, that then made us look at it, which is a good thing, you know, and, but we very much quickly were like, well, we don't just want to kind of comply, i.e., roll an average statement out, we want to make sure we kind of try and grasp this quite strongly.* (Participant 23)

This shows that the legislation has produced changes in companies’ awareness, and in some cases encouraged a sense of responsibility around modern slavery that goes beyond the legal minimum actions MSA demands.

### Complementary and Conflicting Logics

**Fig. 1:**
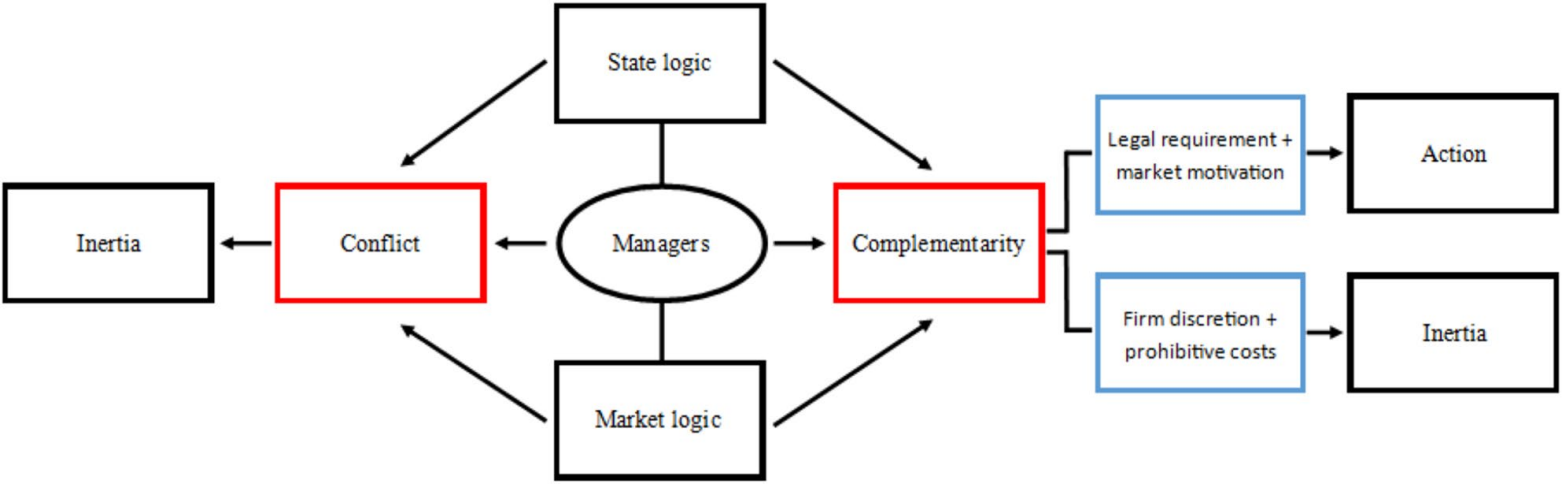
A process model of logic interaction and outcomes

We have demonstrated that both market and state logics are acknowledged by practitioners in our sample as being key drivers for the actions they have put in place in response to MSA. However, in order to understand more clearly how firms’ responses have been influenced by market and state logics, it is necessary to consider how managers view the two logics as interacting. In the following section, we argue that there are both complementary and conflicting elements. How these logics interact, through managers’ understanding and decision making, to produce responses to MSA is illustrated in Fig. 1. Where there is conflict between the logics the outcome is inertia, through managers feeling their ability to act is constrained. Complementarity, on the other hand, can produce two outcomes. First, the coercive legal requirement combines with a market-based motivation, such as reputational concerns, resulting in firms taking action. Second, the combination of the market disincentivising actions beyond those legally required and the discretion MSA gives firms beyond the legal minimum of publishing a modern slavery statement produces a second pathway to inertia. The two sections which follow explain and evidence these dynamics.

### Complementarity

The most immediate point in relation to the logics’ complementarity is that, as outlined above, market and state logics both clearly encourage firms to develop a response to modern slavery risks. Responding to MSA is viewed as necessary for the business and desirable from an ethical standpoint. This is a product of the risk of reputational damage, competitor behaviour, and clients’ requirements within the market logic, and the legal obligation and normative pressures understood by our respondents in the state logic. Consequently, it is possible to assert that there is a positive case for addressing modern slavery. As one Senior Procurement Manager put it.

*I think absolutely with regards to the work that you can do to, to wheedle [modern slavery] out to prove to your client at the end of the day that you're offering them a fully secure service that delivers on its corporate social responsibility but delivers on the fact that they are doing everything they can to preserve their relationship, their reputation, and ultimately that of the client, then it’s a huge selling point.* (Participant 17)

In other words, complementarity between the two logics is seen in the alignment between both the normative and legal aspects of the state logic and the business case for doing so within the market logic. Consequently, there is no disagreement over the question of whether to comply with MSA, and firms often chart their journey in addressing modern slavery risks from the passing of the Act onwards. There is therefore little doubt that complementarity between the two logics has produced compliance with MSA.

However, the second area of complementarity between the market and state logics effectively places a ceiling on how much the alignment between the two logics can achieve. This aspect of complementarity is found in the fact that the state logic also contains an element of market logic: actions beyond compliance with MSA are expected to be brought about by market forces, creating a race to the top for transparency and anti-slavery measures. This means responses to MSA are discretionary, and in this context the market and the state logics can align to produce very little change beyond the production and publication of modern slavery statements, hence the inertia pathway detailed in Fig. 1. As a Supply Chain Director pointed out, managers will seek to meet legal obligations, but not go beyond them: “*if I'm being honest, it's still the case of within our industry of tell us what the minimum is and we’ll kind of do that until you force upon us to do more*” (Participant 14). A key point is that once all principal contractors have achieved compliance with MSA, they are able to defend against the risk of reputational damage because they can accurately claim to have met their legal obligations as well as client requirements, and to have in place the same measures as their competitors. As such, those pressures are effectively neutralized. A Senior Procurement Manager summarized the result of this dynamic for how the industry has responded to MSA:

*All we’ve created is a cottage industry, creating a piece of paper that effectively acknowledges that modern slavery exists, I’ve ticked the box, I’ve done the bare minimum and I’m great thanks.* (Participant 17)

Complementarity between the market elements of both logics has therefore compelled managers to aim for compliance as a minimum, but it has also placed a ceiling on what is likely to be achieved.

### Conflict

Despite the complementarity between the two logics leading to weak responses, there is nevertheless a remaining conflict between the logics due to the normative aspects of the state logic placing expectations on managers to address modern slavery risks, which comes into conflict with various elements of the market logic.

One area of conflict is between the expectation and desire to address modern slavery risks and the perceived costs of implementing more substantive anti-slavery measures. As one Social Sustainability Manager put it,

*We don’t want to compete on human rights but there is a cost to implementing a proper ethical labour sourcing type of strategy or approach with plans that tackle every area logically and concisely.* (Participant 15)

Consequently, there is a trade-off in managers’ decision making which places the normative aspect of the state logic against prioritizing competitiveness. A similar point was made by a Procurement Leader: “*we simply won’t win any work because yes, we do the right thing but we're twice as expensive as everyone else”* (Participant 2). In other words, it is recognized that to enact an ethical approach to modern slavery where substantial new measures were put in place is to actively operate against the logic of profitability. It is not only that implementing changes to existing practices is viewed as incurring costs; but rather that this may in fact harm a company’s position in the market.

*From an absolute competitive point of view, to some degree [developing further measures to manage modern slavery risk] gives you a disadvantage because you’ve probably got to take on more costs… The more risk-averse you are, the more cost you might take on compared with a less risk-averse organization*. (Participant 18)

In other words, other firms that are doing less to address modern slavery risks are able to maintain lower costs. The view is that these firms are more likely to be awarded work on this basis, particularly if those firms are meeting the standards clients require.

Another area of conflict between market and state logics involves the role clients play in establishing funds available for addressing modern slavery risk, or, more precisely, where they do not.

*I think it has to come from the client, if you want to do it and make money, it's very hard really because, like I said, you know, profit does go on collision course with some of these other things at times, you know, and there's no getting away from that, it absolutely does, you know, so when the client wants it, it's easier.* (Participant 1)

For one Senior Sustainability Manager this conflict became quite literal as they attempted to negotiate with a client for additional funds in order to more substantially address modern slavery risks, but their efforts were rebuffed.

*I guess at the minute the consequences aren’t being seen as significant enough to put in the up-front money… modern slavery you might say it’ll be an extra 50 grand, it’s, it’s not, it’s not massive amounts, you know, to make sure this doesn’t happen on your site. That’s it. That’s just what, we were just asking, but [the client’s] saying, “well, you shouldn’t have modern slaves on your site anyway, should you?”. It’s like, well no but we know it exists, so it’s kind of like, it’s a bit of a difficult conversation.* (Participant 28)

This not only highlights the key role clients can play in making resource available for contractors to tackle issues such as modern slavery, but also the different ways in which conflict between the two logics can manifest.

Finally, conflict is also found between the market and state logics within the state itself, which is both the producer of MSA and a client to the construction industry. A Corporate Responsibility Director pointed out the challenge of attempting to persuade the Government to move away from a low-cost procurement approach:

*You'd just be surprised with the leadership approach from Government if I'm perfectly honest, that it is about price. And we have to try, sometimes quite hard, to be able to get them to flex on best value rather than lowest price. And where I see a risk in how government procure is where they have intermediary organisations managing contracts on behalf of Government who are incentivized on delivering price.* (Participant 10)

In other words, the Government produce modern slavery legislation with one hand, and contribute to the challenges that contractors face in addressing modern slavery with the other.

## Discussion

In this article, we offer insights into the competing pressures firms come under in responding to modern slavery legislation and the supply chain risks of modern slavery. Specifically, we study the conflicting institutional logics within the UK construction industry. We find in our data a breadth of attitudes to the legislation, from catalyst of awareness to the tick box results, to reputational concern, to the desire to minimize the risks of slavery on construction sites. Overarching these reactions, we find that the market and state logics operate in both complementary and conflictual ways. Through the complementarity between market aspects of both logics, compliance with the legislation is produced, but little else. Conflict between the market logic and normative expectations created by the state logic has led to an irreconcilable tension between mangers’ desire to act responsibly on one hand, and the need to operate profitability and maintain competitiveness on the other.

Our study makes three contributions: to the phenomenological literature on modern slavery; to theory, in institutional logics; and to the construction industry literature. First, across disciplines the literature on modern slavery focusses on the levels of compliance (Stevenson & Cole, 2018), the determinants thereof (Flynn, 2020), or other aspects of disclosure in response to MSA (Schaper & Pollach, 2021). The implied assumption in this theme is that compliance with MSA is the goal. Compliance with MSA is not the goal. The goal of the law is “to undertake fundamental steps towards identifying risks in their own supply chains and developing suitable and effective due diligence processes” (Martin-Ortega & Krupinska, 2018). While Rogerson et al. (2020) find a focus on compliance and an absence of board-level interest stifling public sector efforts to address modern slavery, little work has been done to investigate the tensions which occur as firms attempt to address modern slavery risk. We contribute in this respect by using institutional logics to explain how the various pressures on construction industry managers affect decision making around modern slavery.

Our analysis explains, rather than assumes, the key role the market plays in shaping responses to modern slavery risk and MSA. For example, Flynn (2020) explains the aim of compliance as being social legitimacy but does not account for the role the market plays in bringing this about. Similarly, Gutierrez-Huerter O et al. (2021) discuss the ways in which responses to MSA have been framed over time among competing actors, but do not directly address market conditions as a factor that explains company responses. In short, our study addresses a deficit in the modern slavery literature around the key role the market—alongside the state—has played in explaining how companies approach the presence of modern slavery.

Through the state logic, and the normative expectations it has created through raising awareness, we are also able to highlight the role of individuals’ moral motivations in how companies develop responses to modern slavery. For example, Monciardini et al. (2021) argue that companies resist making changes to existing practices through managerializing law to ensure minimal change in business practices. We find this to be overly dismissive of individuals’ anti-slavery commitment. Our findings support the view that it is often key individuals that drive forward change. As Carter and Jennings (2004, p.154) put it, the “personal values of employees have been espoused as one potential precursor to ethical behaviour in organizations”. However, the dominance of the market logic over the normative element of the state logic shows that managers who are desirous of achieving change feel powerless to do so when that organizational change is viewed as undermining profitability.

Second, we contribute to the institutional logics literature. In doing so, we introduce logics to the modern slavery literature, answering a call from Phung and Crane (2018) to advance our understanding of modern slavery in organizations through perception and interpretation of the underlying pressures of organizational action around the phenomenon. The literature on institutional logics understandably focusses on a market logic (Besharov & Smith, 2014; Dahlmann & Grosvold, 2017; Goodrick & Reay, 2011). The market logic is often found to compete with other established and emerging logics (McLoughlin & Meehan, 2021; Nicholls & Huybrechts, 2016). Recently, research has begun to find the dynamism of institutional settings has brought about an uneasy conciliation between logics (Dahlmann & Grosvold, 2017; Lounsbury et al., 2021). Beyond the context of modern slavery, we contribute to the institutional logics literature by providing a new way to understand how two logics can interact to produce organizational outcomes: simultaneously through forms of complementarity and conflict. Our model, illustrated in Fig. 1, provides a way to understand how pressures produced by logics can develop into specific pathways—leading either to action or inertia—for decision makers, in our context managers. This model could be applied to other contexts using the state and market logics, or it could be used to explain complementarity and conflict found between logics other than market and state.

We might anticipate that legislation which runs counter to the market logic might promote such a conciliatory state given the centrality of the law to institutional change (DiMaggio & Powell, 1983). However, we find that the market logic works in harmony with the state logic in one sense (at minimum leading to legal compliance) and dominates it in another (to subordinate further anti-slavery measures). Prior studies have found that state intervention is an important factor in altering the interplay between logics (Greenwood et al., 2010; Reay & Hinings, 2005). We problematize the role of government as counterintuitively key in the development of new logics around modern slavery. Extant studies generally find that when the state has mandated changes to an institutional field, bringing about a logic which contends with the market logic (e.g. Reay & Hinings, 2005), the market logic dominates. We find, however, that the state’s unwillingness to use its coercive influence runs counter to the tenets of institutional theory more generally (DiMaggio & Powell, 1983) and institutional logics more specifically (Greenwood et al., 2010). Not only is the state disinclined to back legislation with action, its dual role in our context as legislator and as client means that the contradictory roles of government as enforcer of law and as consumer undermine the conciliation of market and state logics. Logics seen in beliefs held by individual managers, which have been found elsewhere to dictate socially responsible practices (e.g. Wickert et al., 2017) and which we see in our data in the state logic, are therefore prevented from developing to realistically vie with the market logic.

This raises the question which is not new in discussions of modern slavery and MSA, of the efficacy of using market mechanisms in attempts to drive better organizational behaviour. We provide, from an institutional logics perspective and from empirical evidence gathered from firm responses to modern slavery, support for prior views questioning the state’s use of the market logic (e.g. Harris & Nolan, 2022; LeBaron, 2020). The scepticism expressed by LeBaron and Rühmkorf (2017: 26) that “the substitution of a vague reporting requirement over a more stringent model of public governance appears to have undermined MSA’s effectiveness in ‘steering’ corporate behaviour” is thus borne out. The state’s failure to mobilize its coercive power to change firm behaviour has resulted, in the words of one of our respondents, in the creation of little more than a “cottage industry, creating a piece of paper that effectively acknowledges that modern slavery exists”.

Though we find conciliation, we also identify an irreconcilable conflict between the market logic and aspects of the state logic which speak to the desire for action in line with the spirit of MSA. While the logics literature maintains that competition between logics can be ongoing (e.g. McLoughlin & Meehan, 2021; Nicholls & Huybrechts, 2016; Reay & Hinings, 2009), prior research has taken for granted that a logic will dominate, the other(s) becoming subservient. But while these other logics remain present, the ‘contest’ is ‘won’ and settled by the dominant logic. We highlight that friction between logics can be continuous where two logics cannot be reconciled, at least in some respects, suggesting not that one logic becomes subservient but that it can be the source of ongoing frustrations.

Third, we contribute to the literature on the construction industry. The power of the market logic illustrates the challenges even the most ethically minded practitioners face in attempting to address modern slavery. The conflict we identify between market and state logics prevents adequate actions from being taken to mitigate modern slavery risk in labour supply. As such, our findings have implications for ethical procurement. As Smit et al., (2021, p.9) note, “in order to be effective, codes of conduct also need to be complemented by purchasing practices which similarly take account of human rights”. In other words, bringing about changes in procurement are necessary to further reduce modern slavery risk, but, as our analysis shows, practitioners do not believe they have the ability to alter procurement practices in any substantive or meaningful way without negatively impacting their ability to compete for work.

Our findings and analysis also contradict views held elsewhere that bringing about ethical changes to the construction industry is likely to occur through the market if clients adopt appropriate practices. For instance, Mustow (2006, p.17) argues that “ultimately, if property and infrastructure buyers and users apply ethical criteria, change will be brought about in the long run through market forces”. However, clients are not providing additional resource for contractors, but instead expect them to enact change within their existing budgets. Thus, market conditions are placing constraints on what contractors are able to do, rather than empowering them. The same point applies to the UK Government when acting as a client to the construction industry. It cannot claim to be taking modern slavery seriously while it is operating on low-cost tender principles, contributing to the challenges contractors face in addressing modern slavery risks.

## Implications and Conclusions

We know from the literature (Christ & Burritt, 2021; Simpson et al., 2021) and from our data that modern slavery is a real and increasing risk to organizations. This is particularly true of the construction industry (Gutierrez-Huerter O et al., 2021; Trautrims et al., 2021). Modern slavery continues to be seen as a far-off problem buried in supply chains, however (LeBaron, 2021; Meehan & Pinnington, 2021; Simpson et al., 2021). While it is undoubtedly the case that the complexity of firm outsourcing arrangements hides modern slavery far from where focal firms make decisions (Bhakoo & Meshram, 2021), multifaceted risks can also be found much closer to home.

Our work focusses on the construction industry’s response to the UK Modern Slavery Act 2015 and modern slavery risks to firms in the industry. Our analysis shows that the competing pressures on firms in the industry have led to a situation in which economic considerations trump responsibility for monitoring and enforcing labour rights issues. We therefore develop recommendations for executives, policymakers, and public sector procurement.

First, executives should come together to work with government to create solutions to the problems we highlight. Solutions will come with costs, but these could be hypothecated to prevent underspend by government and profiteering in industry and could collectively address the issues we raise which are known in the construction industry. While this seems like an obvious step, only one construction company engaged in the public consultation on the independent review of MSA in 2018 (Field MP, 2019). Additionally, working through industry partnerships could contribute towards raising the floor of the minimum actions all firms take. In order to counteract the market pressures we identify in our analysis, actions need to be industry-wide rather than specific to individual firms. Efforts have been made through industry partnerships, but to date this has not been sufficiently cohesive or comprehensive. Second, as Martin-Ortega argues (2017, p.515), “public buyers are as exposed to risks of encountering offences in supply chains as private buyers are. But… while corporations have a responsibility to mitigate the risk and prevent human rights violations in their supply chain, public buyers’, as organs of the state, have heightened obligations in this regard”. If the state therefore continues to refuse to use its coercive power to enact stronger legislation and enforce higher standards, and instead insists upon a market approach, it must improve industry performance by leading in demanding action from construction contractors and in its own annual reporting. The UK government has taken the first step by publishing its modern slavery statement in 2020; the next step is to implement at least the same procurement standards on modern slavery as the best performing private sector clients and contractors in construction.

Our paper has implications beyond its immediate industry context. Understanding the multiple, dynamic institutional pressures on organizations, which impact on how managers understand and react to modern slavery risks, is key in developing an appreciation of factors influencing firm behaviour, which studies repeatedly find is underwhelming across industries on modern slavery. Risks to people working on-site, rather than in supply chains, have already been identified in other industries which rely on manual labour. Where profits are slim, state involvement is high, or labour is outsourced, our findings demonstrate that a market logic can overpower other logics to the detriment of firm accountability and, in the case of modern slavery, human wellbeing. This also therefore has consequences for the idea of a race to the top for ethical ‘slavery-free’ supply chains, as found in government guidance, and comments by advocates of MSA such as Theresa May and the first Anti-Slavery Commissioner, Kevin Hyland. In light of our findings and analysis, there is no reason to believe the market, which presents such significant challenges for addressing modern slavery, is also going to liberate those in exploitative working conditions.

Though our study consists of a wide range of interviews with many of the UK’s largest construction firms, further research is required to ascertain just how generalizable our findings are beyond the context of the UK construction industry. Scholarly work is therefore required in other industries and contexts involving public buyers of manual services. Further work is also needed to identify emerging best practice in the construction industry in the UK and abroad with regard to modern slavery.

## Appendix 1

**Table:**

Participant	Job title	Company type	Firm size (turnover)	Duration of interview (mins)
1	Head of central procurement	Infrastructure	Medium	52
2	Procurement leader	Infrastructure and commercial	Large	61
3	Procurement and supply chain director	Infrastructure and commercial	Medium	38
4	Social value manager	Infrastructure	Medium	56
5	Group procurement manager	Infrastructure and commercial	Large	72
6	National social impact manager	Infrastructure	Large	56
7	Social sustainability manager	Infrastructure and commercial	Large	56
8	Procurement leader	Infrastructure and commercial	Large	45
9	Sustainable procurement manager	Infrastructure	Large	45
10	Corporate responsibility director	Infrastructure	Large	49
11	Director of procurement and supply chain	Infrastructure and commercial	Large	52
12	Head of supply chain	Commercial	Large	42
13	Head of procurement	Infrastructure	Small	47
14	National supply chain director	Commercial	Large	46
15	Social sustainability manager	Infrastructure and buildings	Medium	46
16	Head of procurement	Commercial	Medium	73
17	Senior procurement manager	Infrastructure and commercial	Large	65
18	Group Procurement Director	Housing	Small	50
19	Group head of supply chain	Infrastructure	Large	45
20	Senior supply chain manager	Infrastructure	Large	55
21	Group procurement director	Commercial	Large	40
22	Head of procurement (Europe)	Infrastructure and commercial	Medium	46
23	Head of ethical and sustainable procurement	Infrastructure and commercial	Large	47
24	Responsible business director	Commercial	Large	51
25	Procurement director	Infrastructure	Small	62
26	Director of sustainability and procurement	Infrastructure and commercial	Large	41
27	Group commercial & compliance director	Infrastructure and commercial	Small	78
28	Senior sustainability manager	Commercial	Large	42
29	Head of employee relations	Infrastructure and commercial	Large	58
30	Head of sustainability	Commercial	Small	43

## References

[CR1] Aberbach JD, Rockman BA (2002). Conducting and coding elite interviews. Political Science and Politics.

[CR2] Alberti G, Danaj S (2017). Posting and agency work in British construction and hospitality: The role of regulation in differentiating the experiences of migrants. The International Journal of Human Resource Management.

[CR3] Ali, B. (2018). Bogus workers and the hidden threat of site card fraud. *Construction News*. Available at: https://www.constructionnews.co.uk/news/knowledge-news/bogus-workers-and-the-hidden-threat-of-site-card-fraud-15-11-2018/ Accessed 9 October 2021.

[CR4] Arena M, Azzone G, Mapelli F (2018). What drives the evolution of corporate social responsibility strategies? An institutional logics perspective. Journal of Cleaner Production.

[CR5] Behling F, Harvey M (2015). The evolution of false self-employment in the British construction industry: A neo-Polanyian account of labour market formation. Work, Employment and Society.

[CR6] Benstead AV, Hendry LC, Stevenson M (2018). Horizontal collaboration in response to modern slavery legislation: An action research project. International Journal of Operations and Production Management.

[CR7] Besharov ML, Smith WK (2014). Multiple institutional logics in organizations: Explaining their varied nature and implications. Academy of Management Review.

[CR8] Bhakoo V, Meshram K, Oritzsky M, Pless N, Sandhu S, Maak T (2021). Modern slavery in supply chains. The Routledge Companion to Corporate Social Responsibility.

[CR9] Bhatia-Lin A, Boon-Dooley A, Roberts MK, Pronai C, Fisher D, Parker L, Engstrom A, Ingraham L, Darnell D (2019). Ethical and regulatory considerations for using social media platforms to locate and track research participants. The American Journal of Bioethics.

[CR10] BHRRC. (2017). First year of FTSE 100 reports under the UK Modern Slavery Act: Towards Elimination? Available at: https://media.business-humanrights.org/media/documents/files/FTSE_100_Report_FINAL_0021Dec2017.pdf Accessed 5 January 2022.

[CR11] BHRRC. (2021) Modern Slavery Act: Five years of reporting. Conclusions from monitoring corporate disclosure. Available at: https://media.business-humanrights.org/media/documents/MSR_Embargoed.pdf Accessed 5 January 2022.

[CR12] Boersma M, Nolan J (2022). Modern slavery and the employment relationship: Exploring the continuum of exploitation. Journal of Industrial Relations.

[CR13] Bouncken RB, Qiu Y, Sinkovics N, Kürsten W (2021). Qualitative research: Extending the range with flexible pattern matching. Review of Managerial Science.

[CR14] Braun V, Clarke V (2021). To saturate or not to saturate? Questioning data saturation as a useful concept for thematic analysis and sample-size rationales. Qualitative Research in Sport, Exercise and Health.

[CR15] Campbell JL (2006). Institutional analysis and the paradox of corporate social responsibility. American Behavioral Scientist.

[CR16] Carter CR, Jennings MM (2004). The role of purchasing in corporate social responsibility: A structural equation analysis. Journal of Business Logistics.

[CR17] Caruana R, Crane A, Gold S, LeBaron G (2021). Modern slavery in business: The sad and sorry state of a non-field. Business & Society.

[CR18] CBI, (2020). Fine margins: Delivering financial sustainability in UK construction. Available at: https://www.cbi.org.uk/articles/fine-margins-delivering-financial-sustainability-in-uk-construction-bv/ Accessed 8 October 2021.

[CR19] Christ KL, Burritt RL (2021). Accounting for modern slavery risk in the time of COVID-19: Challenges and opportunities. Accounting, Auditing & Accountability Journal.

[CR20] CITB, n.d. Fraudulent construction scheme cards. Available at: https://www.citb.co.uk/courses-and-qualifications/check-a-card-training-record/fraudulent-cards/ Accessed 9 October 2021.

[CR21] Clarke L (2006). Valuing labour. Building Research & Information.

[CR22] Corbett J, Webster J, Jenkin T (2018). Unmasking corporate sustainability at the project level: Exploring the influence of institutional logics and individual agency. Journal of Business Ethics.

[CR23] Cousins P, Dutordoir M, Lawson B, Neto JQF (2020). Shareholder wealth effects of modern slavery regulation. Management Science.

[CR24] Crane A (2013). Modern slavery as a management practice: Exploring the conditions and capabilities of human exploitation. Academy of Management Journal.

[CR25] Crates, E. (2018). Construction and the Modern Slavery Act: Tackling exploitation in the UK. Available at: https://www.ciob.org/sites/default/files/Construction%20and%20the%20Modern%20Slavery%20Act_0.pdf Accessed 9 October 2021.

[CR26] Dahlmann F, Grosvold J (2017). Environmental managers and institutional work: Reconciling tensions of competing institutional logics. Business Ethics Quarterly.

[CR27] DiMaggio PJ, Powell WW (1983). The iron cage revisited: Institutional isomorphism and collective rationality in organizational fields. American Sociological Review.

[CR28] Ergon. 2018. Modern slavery reporting: Is there evidence of progress? Available at: https://ergonassociates.net/wp-content/uploads/2018/10/Ergon_Modern_Slavery_Progress_2018_resource.pdf Accessed 5 January 2022.

[CR29] Field MP, Rt. Hon. F. (2019). Independent review of the Modern Slavery Act: Final report. Available from: https://www.gov.uk/government/publications/independent-review-of-the-modern-slavery-act-final-report/independent-review-of-the-modern-slavery-act-final-report-accessible-version [accessed 3 August 2022].

[CR30] FLEX. (2018) *Shaky Foundations: Labour Exploitation in London’s Construction Sector*, Focus on Labour Exploitation, pp. 1–26

[CR31] Flynn A (2020). Determinants of corporate compliance with modern slavery reporting. Supply Chain Management: An International Journal.

[CR32] Flynn A, Walker H (2021). Corporate responses to modern slavery risks: An institutional theory perspective. European Business Review.

[CR33] Friedland R, Alford RR, Powell WW, DiMaggio PJ (1991). Bringing society back in. The New Institutionalism in Organizational Analysis.

[CR34] Garbers K (2022). Unseen Lives: The Hidden World of Modern Slavery.

[CR35] Garner-Purkis, Z. (2019) Revealed: Sites where modern slavery victims worked, *Construction News*, available: https://www.constructionnews.co.uk/news/revealed-sites-modern-slavery-victims-worked-16-09-2019 Accessed 4 July 2022.

[CR36] Gelinas L, Pierce R, Winkler S, Cohen IG, Lynch HF, Bierer BE (2017). Using social media as a research recruitment tool: Ethical issues and recommendations. The American Journal of Bioethics.

[CR37] Gillham B (2007). Research interviewing: The range of techniques.

[CR38] GLAA. (2020). Gangmasters and labour abuse authority: Annual report and accounts. Crown copyright. Available at: https://assets.publishing.service.gov.uk/government/uploads/system/uploads/attachment_data/file/960077/GLAA_Annual_Report_and_Accounts_2019-20_e-laying_version_10.02.21_dated_7_January.pdf Accessed September 12 2021.

[CR39] Glavas A, Mish J (2015). Resources and capabilities of triple bottom line firms: Going over old or breaking new ground?. Journal of Business Ethics.

[CR40] Gold S, Trautrims A, Trodd Z (2015). Modern slavery challenges to supply chain management. Supply Chain Management: An International Journal.

[CR41] Goodrick E, Reay T (2011). Constellations of institutional logics: Changes in the professional work of pharmacists. Work and Occupations.

[CR42] HM Gov (2015) *Transparency in Supply Chains etc. A practical guide*. Available at: https://assets.publishing.service.gov.uk/government/uploads/system/uploads/attachment_data/file/1040283/Transparency_in_Supply_Chains_A_Practical_Guide_2017_final.pdf Accessed 1 July 2022.

[CR43] Greenwood R, Díaz AM, Li SX, Llorente JC (2010). The multiplicity of institutional logics and the heterogeneity of organizational responses. Organization Science.

[CR44] Gümüsay AA, Claus L, Amis J (2020). Engaging with grand challenges: An institutional logics perspective. Organization Theory.

[CR45] Gutierrez-Huerter O, G., Gold, S., & Trautrims, A. (2021). A change in rhetoric but not in action? Framing of the ethical issue of modern slavery in a UK sector at high risk of labour exploitation. *Journal of Business Ethics*. 10.1007/s10551-021-05013-w PMC8649994 34898767

[CR46] Harris H, Nolan J (2022). Outsourcing the enforcement of modern slavery: Overcoming the limitations of a market-based disclosure model. Journal of Industrial Relations.

[CR47] Hartmann A, Caerteling J (2010). Subcontractor procurement in construction: The interplay of price and trust. Supply Chain Management: An International Journal.

[CR48] ILO. (2001). *The construction industry in the twenty-first century: Its image, employment prospects and skill requirements*, International Labour Organisation. Available at: https://www.ilo.org/global/publications/ilo-bookstore/order-online/books/WCMS_PUBL_9221126226_EN/lang--en/index.htm Accessed 27 July 2022.

[CR49] Islam MA, Van Staden CJ (2021). Modern slavery disclosure regulation and global supply chains: Insights from stakeholder narratives on the UK Modern Slavery Act. Journal of Business Ethics.

[CR50] Janghorban R, Roudsari RL, Taghipour A (2014). Skype interviewing: The new generation of online synchronous interview in qualitative research. International Journal of Qualitative Studies on Health and Well-Being.

[CR51] Jones P, Comfort D (2022). Modern slavery statements and the UK’s largest housebuilding companies: An exploratory research paper. Property Management, at Press,.

[CR52] Kriebitz A, Max R (2020). The Xinjiang case and its implications from a business ethics perspective. Human Rights Review.

[CR53] Lancaster K (2017). Confidentiality, anonymity and power relations in elite interviewing: Conducting qualitative policy research in a politicised domain. International Journal of Social Research Methodology.

[CR54] LeBaron G (2020). Combatting Modern Slavery.

[CR55] LeBaron G (2021). The role of supply chains in the global business of forced labour. Journal of Supply Chain Management.

[CR56] LeBaron G, Ruhmkorf A (2017). Steering CSR through home state regulation: A comparison of the impact of the UK Bribery Act and Modern Slavery Act on global supply chain governance. Global Policy.

[CR57] LeBaron G, Rühmkorf A (2019). The domestic politics of corporate accountability legislation: Struggles over the 2015 UK Modern Slavery Act. Socio-Economic Review.

[CR58] Lee MP, Lounsbury M (2015). Filtering institutional logics: Community logic variation and differential responses to the institutional complexity of toxic waste. Organization Science.

[CR59] Lounsbury M, Steele CWJ, Wang MS, Toubiana M (2021). New directions in the study of institutional logics: From tools to phenomena. Annual Review of Sociology.

[CR60] Manu E, Knight A, Pryke S (2020). Understanding supply chain management from a main contractor’s perspective. Successful Construction Supply Chain Management: Concepts and Case Studies.

[CR61] Martin-Ortega O (2017). Human rights risks in global supply chains: Applying the UK Modern Slavery Act to the public sector. Global Policy.

[CR62] Martin-Ortega, O., & Krupinska, P. (2018). UK modern slavery act transparency in supply chains: The second year of reporting by universities. Available at: http://www.bhre.org/blog/2018/3/29/transparency-in-university-supply-chains-bhre-comparative-report Accessed 15 November 2019.

[CR63] Mason J (2018). Qualitative Researching.

[CR64] May, T. (2016). My government will lead the way in defeating modern slavery. *The Telegraph*, 30 July 2016. Available at: https://www.telegraph.co.uk/news/2016/07/30/we-will-lead-the-way-in-defeating-modern-slavery/ Accessed 12 September 2021.

[CR65] McLoughlin K, Meehan J (2021). The institutional logic of the sustainable organisation: The case of a chocolate supply network. Institutional Journal of Operations & Production Management.

[CR66] Meehan J, Pinnington BD (2021). Modern slavery in supply chains: Insights through strategic ambiguity. International Journal of Operations & Production Management.

[CR67] Monciardini D, Bernaz N, Andhov A (2021). The organizational dynamics of compliance with the UK Modern Slavery Act in the food and tobacco sector. Business & Society.

[CR68] Mustow SE (2006). Procurement of ethical construction products. Engineering Sustainability.

[CR69] New S (2015). Modern slavery and the supply chain: The limits of corporate social responsibility?. Supply Chain Management: An International Journal.

[CR70] Nicholls A, Huybrechts B (2016). Sustaining inter-organizational relationships across institutional logics and power asymmetries: The case of fair trade. Journal of Business Ethics.

[CR71] Ocasio W, Loewenstein J, Nigam A (2015). How streams of communication reproduce and change institutional logics: The role of categories. Academy of Management Review.

[CR72] Pala M, Edum-Fotwe F, Ruikar K, Doughty N, Peters C (2014). Contractor practices for managing extended supply chain tiers. Supply Chain Management: An International Journal.

[CR73] Palazzo G, Scherer AG (2006). Corporate legitimacy as deliberation: A communicative framework. Journal of Business Ethics.

[CR74] Palinkas LA, Horwitz SM, Green CA, Wisdom JP, Duan N, Hoagwood K (2015). Purposeful sampling for qualitative data collection and analysis in mixed method implementation research. Administration and Policy in Mental Health and Mental Health Services Research.

[CR75] Pesterfield C (2021). Unfree labour and capitalist state: An open Marxist analysis of the 2015 Modern Slavery Act. Capital and Class.

[CR76] Phillips, A., & Trautrims, A. (2018). Agriculture and Modern Slavery Act reporting: Poor performance despite high risks. Available at: http://www.antislaverycommissioner.co.uk/media/1220/modern-slavery-act-and-agriculture-poor-performance-briefing.pdf Accessed 5 January 2022.

[CR77] Phung K, Crane A, Clark J, Poucki S (2018). The business of modern slavery: Management and organizational perspectives. The SAGE handbook of human trafficking and modern day slavery.

[CR78] Pinheiro SM, Emberson C, Trautrims A (2019). ’For the English to see’ or effective change? How supply chains are shaped by laws and regulations and what that means for the exposure of modern slavery. Journal of the British Academy.

[CR79] Pinnington B, Benstead A, Meehan J (2022). Transparency in supply chains (TISC): Assessing and improving the quality of modern slavery statements. Journal of Business Ethics.

[CR80] Reay T, Hinings CR (2005). The recomposition of an organizational field: Health care in Canada. Organization Studies.

[CR81] Reay T, Hinings CR (2009). Managing the rivalry of competing institutional logics. Organization Studies.

[CR82] Reay T, Jones C (2016). Qualitatively capturing institutional logics. Strategic Organization.

[CR83] Rioux S, LeBaron G, Verovšek PJ (2020). Capitalism and unfree labour: A review of Marxist perspectives on modern slavery. Review of International Political Economy.

[CR84] Rogerson M, Crane A, Soundararajan V, Grosvold J, Cho CH (2020). Organisational responses to mandatory modern slavery disclosure legislation: A failure of experimentalist governance?. Accounting, Auditing & Accountability Journal.

[CR85] Saunders B, Sim J, Kingstone T, Baker S, Waterfield J, Bartlam B, Burroughs H, Jinks C (2018). Saturation in qualitative research: Exploring its conceptualization and operationalization. Quality & Quantity.

[CR86] Savarese C, Huybrechts B, Hudon M (2020). The influence of interorganizational collaboration on logic conciliation and tensions within hybrid organizations: Insights from social enterprise–corporate collaborations. Journal of Business Ethics.

[CR87] Schaper S, Pollach I (2021). Modern slavery statements: From regulation to substantive supply chain reporting. Journal of Cleaner Production.

[CR88] Simpson D, Segrave M, Quarshie A, Kach A, Handfield R, Panas G, Moore H (2021). The role of psychological distance in organizational responses to modern slavery risk in supply chains. Journal of Operations Management.

[CR89] Sinkovics N, Cassell C, Cunliffe AL, Grandy G (2018). Pattern matching in qualitative analysis. The SAGE Handbook of Qualitative Business and Management Research Methods: Methods and Challenges.

[CR90] Smit L, Holly G, McCorquodale R, Neely S (2021). Human rights due diligence in global supply chains: Evidence of corporate practices to inform a legal standard. The International Journal of Human Rights.

[CR91] Spino J (2019). Research participant communication via social media platforms remains risky. The American Journal of Bioethics.

[CR92] Stevenson M (2022). Hidden in plain sight: The bystander effect and the mobilisation of modern slavery whistleblowing. Supply Chain Management: An International Journal.

[CR93] Stevenson M, Cole R (2018). Modern slavery in supply chains: A secondary data analysis of detection, remediation and disclosure. Supply Chain Management: An International Journal.

[CR94] Taylor, M., & Addley, E. (2008). Migrant builder took home £8.80 for a week, *The Guardian*. Available at: https://www.theguardian.com/politics/2008/jun/30/tradeunions.pay Accessed 27 September 2021.

[CR95] Thornton PH, Ocasio W (1999). Institutional logics and the historical contingency of power in organizations: Executive succession in the higher education publishing industry, 1958–1990. American Journal of Sociology.

[CR96] Thornton PH, Ocasio W, Lounsbury M (2012). The institutional logics perspective: A new approach to culture, structure, and process.

[CR97] Trautrims A, Gold S, Touboulic A, Emberson C, Carter H (2021). The UK construction and facilities management sector’s response to the Modern Slavery Act: An intra-industry initiative against modern slavery. Business Strategy and Development.

[CR98] Unseen (2019). Impact report 2019. Available at: https://www.unseenuk.org/support-us/businesses/forced-labour-in-the-uk Accessed 12 September 2021.

[CR99] Van Buren HJ, Schrempf-Stirling J, Westermann-Behaylo M (2021). Business and human trafficking: A social connection and political responsibility model. Business & Society.

[CR100] Voss H, Davis M, Sumner M, Waite L, Ras IA, Singhal D, Jog D (2019). International supply chains: Compliance and engagement with the Modern Slavery Act. Journal of the British Academy.

[CR101] Waldorff SB, Reay T, Goodrick E, Lounsbury M, Boxenbaum E (2013). A tale of two countries: How different constellations of logics impact action. Institutional Logics in Action, Part A.

[CR102] Weinfass, I. (2021). 13 arrested over modern slavery offences, *Construction News*. Available at: https://www.constructionnews.co.uk/legal/13-arrested-in-uk-and-romania-over-modern-slavery-offences-29-09-2021/ Accessed 11 January 2022.

[CR103] Westermann-Behaylo M, Berman SL, Van Buren III, H. J. (2014). The influence of institutional logics on corporate responsibility toward employees. Business & Society.

[CR104] Wickert C, Vaccaro A, Cornelissen J (2017). ‘‘Buying’’ corporate social responsibility: Organisational identity orientation as a determinant of practice adoption. Journal of Business Ethics.

[CR105] Wray-Bliss E, Michelson G (2021). Modern slavery and the discursive construction of a propertied freedom: Evidence from Australian business. Journal of Business Ethics, at Press..

[CR106] Yin J, Jamali D (2021). Collide or collaborate: The interplay of competing logics and institutional work in cross-sector social partnerships. Journal of Business Ethics.

[CR107] Zielinski, P. (2019). Modern slavery in the construction sector. *Chartered Institute of Building*. Available at: https://www.ciob.org/blog/modern-slavery-construction-sector Accessed 9 October 2021.

